# Design of a Platelet-Mediated Delivery System for Drug-Incorporated Nanospheres to Enhance Anti-Tumor Therapeutic Effect

**DOI:** 10.3390/pharmaceutics13101724

**Published:** 2021-10-18

**Authors:** Jun-ichiro Jo, Tsubasa Emi, Yasuhiko Tabata

**Affiliations:** Laboratory of Biomaterials, Institute for Frontier Life and Medical Sciences, Kyoto University, 53 Kawara-cho Shogoin, Sakyo-ku, Kyoto 606-8507, Japan; jo@infront.kyoto-u.ac.jp (J.-i.J.); ind.chemi.tsubasa@gmail.com (T.E.)

**Keywords:** platelets, nanopsheres, drug delivery system, poly(lactic-co-glycolic acid), tumor

## Abstract

The objective of this study is to construct a platelet-mediated delivery system for drug-incorporated nanospheres. Nanospheres of poly(lactic-co-glycolic acid) (PLGA-NS) with different sizes and surface properties were prepared by changing the preparation parameters, such as the type of polymer surfactant, the concentration of polymer surfactant and PLGA, and the stirring rate. When incubated with platelets, PLGA-NS prepared with poly(vinyl alcohol) suppressed the platelet activation. Scanning electron microscopic and flow cytometry examinations revealed that platelets associated with PLGA-NS (platelet hybrids, PH) had a similar appearance and biological properties to those of the original platelets. In addition, the PH with PLGA-NS specifically adhered onto the substrate pre-coated with fibrin to a significantly great extent compared with PLGA-NS alone. When applied in an in vitro model of tumor tissue which was composed of an upper chamber pre-coated with fibrin and a lower chamber culturing tumor cells, the PH with PLGA-NS incorporating an anti-tumor drug were delivered to the tumor cells through the specific adhesion onto the upper chamber and, consequently, drug release from the upper chamber took place, resulting in the growth suppression of tumor cells. It is concluded that the drug delivery system based on PH is promising for tumor treatment.

## 1. Introduction

Malignant tumors continue to be a major problem for the life of human beings. Among the main strategies for tumor treatment, chemotherapy has attracted much attention, because chemotherapy can compensate the shortcomings of surgical tumor treatment, such as tumor recurrent and metastasis. So far, various types of chemotherapeutic agents have been developed and used in clinical settings [1,2,3]. However, most chemotherapeutic agents have no inherent tumor targetability, which causes an adverse effect due to a high dose administration.

To improve the tumor targetability, drug delivery systems using nanospheres have been widely explored [4,5,6]. Since it is comparably easy to adjust the physicochemical properties of nanospheres, the spatial- and temporal-controlled drug delivery can be achieved. It is well recognized that the nanospheres with a diameter less than 100 nm, and the coating with some water-soluble polymers, such as polyethylene glycol and hyaluronic acid, accumulate in the vascular-rich tumor tissue by so-called enhanced permeability and retention effect [7]. In addition, the surface modification with biological ligands enables nanospheres to deliver to the target tissue [8,9]. However, the targetability is still low because the nanospheres are often recognized by the reticuloendothelial system and eliminated from blood circulation in a short time period [10].

Recently, in addition to the materials-based system of drug delivery described above, a new drug targeting strategy by combining cells or their membrane, has increasingly attracted attention due to their inherent abilities, such as the minimal interaction with normal cells, the homing ability for desired cells, and the escape nature from immune surveillance. Various cell types of erythrocytes, leukocytes, platelets, and stem cells have been utilized for this strategy of drug delivery based on the natural cellular components [11,12,13,14,15]. Among them, the combination of platelets and their membrane is one of the most feasible strategies because it is expected that their innate functions can be applied for various vascular disorders (e.g., tumor, inflammation, thrombosis, and hemorrhage) [16,17,18]. It has been reported that there is a close relationship between the tumor malignancy and platelets [19]. Additionally, in tumor tissues, the active tumor invasion into tumor blood vessels causes a number of microhemorrhage (immature fibrin clot) [20,21]. Based on this knowledge, several studies on drug-loading platelets for tumor treatments have been reported, although the drug loaded is limited to only water-soluble drugs [16,17,22].

The platelets physiologically play an important role in the removal of foreign materials from the blood circulation through uptake [23,24]. Based on this characteristic, there have been studies that reported on the internalization of latex particles and liposomes by platelets [23,24,25,26]. In this context, it can be hypothesized here that platelets could be a natural cell carrier for water-insoluble drug-loaded nanospheres aiming at natural targeting of tumor tissues. In the tumor tissues, inflammation reactions take place, leading to the subsequent deposition of fibrin. Platelets have an inherent ability for the adhesion to fibrin. Based on this mechanism, platelets naturally target to the tissues. Considering the application of platelets as the natural carrier of drugs, it should be noted that platelets have a unique characteristic of activation which can be observed by aggregation and degranulation to maintain their biological functions [27,28]. When activated, platelets lose their ability for targeting to the tumor tissue and materials uptake [29]. Therefore, to achieve the platelet application as the carrier of nanospheres loading water-insoluble drugs, it is highly required to design nanospheres which avoid the activation of platelets.

The objective of this study is to construct a platelet-mediated delivery system for nanospheres incorporating water-insoluble drugs (platelet hybrids, PH), which enable drugs to specifically deliver to the target site and gradually release. In the present study, nanospheres of poly(lactic-co-glycolic acid) (PLGA-NS) with different sizes and surface properties were prepared for the optimization of nanospheres preparation conditions to minimize the platelet activation. After the incorporation of coumarin-6 or paclitaxel of a water-insoluble anti-tumor drug into the PLGA-NS, the PH with the PLGA-NS were prepared to evaluate their biological functions. We examined the targetability to fibrin and anti-tumor effect of PH in an in vitro tumor tissue model.

## 2. Materials and Methods

### 2.1. Reagents

Poly(lactic-co-glycolic acid) (PLGA, the lactic acid/glycolic acid molar ratio = 75/25, molecular weight = 20,000 Da), thrombin, and paclitaxel (PTX) were purchased from Wako Pure Chemicals Industries, Ltd., Osaka, Japan. Poly(vinyl alcohol) (PVA, degree of polymerization = 1000, degree of saponification = 86–90%) was kindly supplied from Japan Vam & Poval Co., Ltd., Osaka, Japan. Gelatins with isoelectric points of 5.0 (pI5) and 9.0 (pI9) and the weight-averaged molecular weight of 100,000 Da were kindly supplied from Nitta Gelatin Inc., Osaka, Japan. Cellmatrix^®^ type I-P was purchased from Nitta Gelatin Inc., Osaka, Japan. Fibrinogen and adenosine 5′-diphosphate (ADP) were purchased from Sigma-Aldrich Inc., St. Louis, MO, USA. Bovine serum albumin (BSA) was purchased from Nacalai Tesque Inc., Kyoto, Japan. Coumarin-6 (CMR) was purchased from Tokyo Chemical Industry Co., Ltd., Tokyo, Japan. The reagents were used without further purification.

### 2.2. Preparation of PLGA Nanospheres

PLGA nanospheres (PLGA-NS) were prepared by the conventional nano-precipitation method [30]. The acetone solution (1 mL) containing PLGA was added dropwise to an aqueous solution (10 mL) of polymer surfactant, PVA, pI5, and pI9, followed by stirring to form an oil-in-water emulsion. The emulsion was continuously stirred at room temperature to evaporate the residual acetone. In this study, various PLGA-NS with different sizes and surface properties (Table 1) were prepared by changing various preparation parameters. To change the size of PLGA-NS, the concentrations of PLGA and surfactant polymers, stirring rate, and ionic strength of aqueous phase were changed. Different polymer surfactants of PVA, pI5, and pI9 were used to change the surface properties of PLGA-NS. After evaporation, PLGA-NS were collected by the centrifugation of 14,000 rpm for 30 min at 20 °C and resuspended in double distilled water. The procedure of centrifugation and resuspension was repeated 3 times. Then, the PLGA-NS were lyophilized and stored at 4 °C before use. In the preparation of PLGA-NS incorporating CMR (CMR-PLGA-NS) or PTX (PTX-PLGA-NS), the same preparation parameter and procedure as PVA_200_ (Table 1) was used except for the addition of various amounts of CMR or PTX to acetone solution of PLGA before emulsification.

### 2.3. Characterization of PLGA-NS

The PLGA-NS were suspended in 10 mM phosphate-buffered saline solution (PBS, pH 7.4), and the apparent size of nanospheres was measured by dynamic light scattering (DLS, Zetasizer Nano-ZS, Malvern Instruments Ltd., Worcestershire, UK). On the other hand, the PLGA-NS were dissolved in 10 mM phosphate buffer solution (pH 7.4), and the zeta potential was measured by electrophoresis light scattering (ELS, Zetasizer Nano-ZS).

The amount of CMR incorporated into PVA_200_-PLGA-NS was calculated based on the fluorescent intensity of CMR (dimethyl sulfoxide (DMSO) of solvent as a blank) measured by the fluorescent spectrophotometer (SpectraMax i3x, Molecular Devices Japan Co., Ltd., Tokyo, Japan) with excitation and emission wavelengths of 390 and 470 nm, respectively. The amount of PTX incorporated into PVA_200_-PLGA-NS was measured by the high-performance liquid chromatography (HPLC, Prominence LC-AT20, Shimadzu Corp., Kyoto, Japan) equipped with a reverse phase column of C18-MS-II (15 cm, Nacalai Tesque Inc., Kyoto, Japan). The mobile phase, water and acetonitrile, was delivered at 25 °C at the flow rates of 0.35 and 0.65 mL/min, respectively. The PTX eluted was detected by the ultraviolet detector at the wavelength of 230 nm. The area of each eluted peak was integrated and calculated for the PTX quantification (DMSO of solvent as a blank). The measurements were independently performed three times.

### 2.4. Platelet Isolation

The blood from the vena cava of C57BL/6N mice (10- to 15-week-old, male, Shimizu Laboratory Supplies, Kyoto, Japan) was collected by the syringe containing acid-citrate-dextrose solution B (100 µL), followed by the transfer to a tube containing buffered glucose saline-citrate solution (500 µL). The blood collected was centrifuged at 1600 rpm for 5 min at 20 °C to separate into hemocytes and platelet rich plasma (PRP), then the supernatant containing PRP was collected and centrifuged at 1000 rpm for 5 min at 20 °C to fully remove the hemocytes. Finally, the supernatant was centrifuged at 3600 rpm for 5 min at 20 °C, and platelets were collected in the sediment. The platelets were resuspended in modified Tyrode’s buffer (134 mM NaCl, 2.9 mM KCl, 0.34 mM Na_2_HPO_4_, 1 mM MgCl_2_, 10 mM HEPES, and 5 mM D-glucose, pH 7.4). The number of platelets was counted by using the hemocytometer (WakenBtech Co., Ltd., Kyoto, Japan). The platelets prepared were used for further experiments within 3 h.

### 2.5. Effects of PLGA-NS on Platelet

Each PLGA-NS (100 µg) suspended in 1 mL of modified Tyrode’s buffer was mixed with 10^8^ platelets. After 30 min incubation at 37 °C, the mixture was centrifuged at 3600 rpm for 5 min at 20 °C, and platelet number was counted by the hemocytometer. The ADP and thrombin of agonists which bind platelet surface receptors were used to induce the platelet activation.

### 2.6. Preparation of Platelet Hybrids

PVA_200_-PLGA-NS, CMR-PVA_200_-PLGA-NS or PTX-PVA_200_-PLGA-NS (Table 2) were used for the association with platelet. Various amounts of PLGA nanospheres suspended in 1 mL of modified Tyrode’s buffer were mixed with 10^8^ platelets and incubated for 30 min at 37 °C. The mixture was centrifuged twice at 3600 rpm for 5 min at 20 °C to remove free PLGA-NS and resuspended in modified Tyrode’s buffer to obtain platelet hybrids (PH).

To evaluate the amount of CMR-PVA_200_-PLGA-NS associated with platelets, the PH were lyophilized and dissolved in DMSO. The fluorescence intensity of CMR (DMSO solvent as a blank) was measured by the fluorescence spectrophotometer at excitation and emission wavelengths of 390 and 470 nm, respectively. To evaluate the association behavior in one platelet level, the PH (10,000 counts) associated with different amounts of CMR-PVA_200_-PLGA-NS were analyzed on FACSCanto II flow cytometer and Flowjo software (Becton, Dickinson and Company, Franklin Lakes, NJ, USA).

To visually confirm the internalization of CMR-PVA_200_-PLGA-NS into platelets, the PH prepared with CMR-PVA_200_-PLGA-NS were added to a glass bottom dish. The PH were mildly shaken for 30 min at room temperature and washed twice with modified Tyrode’s buffer, followed by the observation by the confocal laser microscopy IX73/CSU-W1 (OLYMPUS Corp., Tokyo, Japan) after staining the platelets with an anti-CD41/61 antibody (emfret Analytics GmbH & Co. KG., Würzburg, Germany).

To morphologically observe the appearance and aggregating behavior of PH, the PH with PVA_200_-PLGA-NS or PTX-PVA_200_-PLGA-NS were added to the glass bottom dish. After 30 min, the PH were washed twice with modified Tyrode’s buffer at room temperature. Then, the samples were fixed with 2.5 wt% glutaraldehyde for 2 h and washed twice with PBS. Samples were dehydrated in ascending ethanol grades for 15 min each and chemically dried with hexamethyldisilazane (Sigma-Aldrich Inc., St. Louis, MO, USA) overnight. The bottom glass plates were cut out by the ultrasonic cutter (USW-334, Honda Electronics Co. Ltd., Aichi, Japan) and fixed on an aluminum support with carbon-adhesive glue and sputter-coated with Au-Pt (E-1010, Hitachi High Tech. Corp., Tokyo, Japan). The samples were observed using a scanning electron microscopy (SU-3500, Hitachi High Tech. Corp., Tokyo, Japan).

### 2.7. Evaluation of Biological Properties of PH

After the preparation of PH with PVA_200_-PLGA-NS or PTX-PVA_200_-PLGA-NS, the PH (10^7^ platelets in 100 µL suspension) were mixed with 10 µL of thrombin (2 U/mL). After 15 min, the PH were stained with 5 µL each of Phycoerythrin (PE)-labeled anti-CD41/61 antibody and Fluorescein isothiocyanate (FITC)-labeled anti-CD62P antibody (emfret Analytics GmbH & Co. KG., Würzburg, Germany) for further 15 min at dark and room temperature. Then, the PH stained (10,000 counts) were analyzed on FACSCanto II flow cytometer and Flowjo software. The platelets incubated with naked PTX for 30 min at 37 °C were also evaluated to investigate the inhibitory effect of platelet activation by PTX.

### 2.8. Evaluation of PH Affinity for Collagen and Fibrin

The glass bottom dish (Matsunami Glass Ind., Ltd., Osaka, Japan) was coated with different proteins of BSA, collagen (Cellmatrix^®^ type I-P), and fibrinogen. Protein coating was performed by adding 2 mL of PBS solution containing protein (0.1 mg/mL) to the dishes and incubating overnight at 37 °C. In the case of fibrinogen, the dish was washed with PBS twice, followed by adding the thrombin solution (1 U/mL) and incubating for 1 h at 37 °C for the conversion of fibrinogen to fibrin. The PH with CMR-PVA_200_-PLGA-NS were added onto the glass bottom dish pre-coated with each protein. After mild shaking for 30 min at room temperature, the dishes were washed twice with the modified Tyrode’s buffer. The fluorescent images were taken by the fluorescent microscope (BZ-X700, Keyence Corp., Osaka, Japan). To evaluate the fluorescent intensity, six images were taken at random and analyzed using the computer program Image J (NIH Inc., Bethesda, MD, USA). The Integrated Density of whole images (IntDen) was used as the fluorescent intensity for comparison.

For time-lapse imaging, the platelets were further cultured in Roswell Park Memorial Institute (RPMI) 1640 Medium (Thermo Fisher Scientific Inc., Waltham, MA, USA) supplemented with 10 vol% bovine fetal calf serum (FCS, GE Healthcare Life Sciences HyClone Laboratories Inc., Logan, UT, USA) and 1 vol% penicillin and streptomycin (Nacalai Tesque Inc., Kyoto, Japan) at 37 °C in a 5% CO_2_–95% air atmospheric condition. At predetermined time points, the medium was totally collected, and then the fresh medium was added for the investigation of CMR release profile. The amount of CMR released from platelet into medium was measured by the fluorescent spectrophotometer as described above.

### 2.9. Evaluation of Drug Delivery by PH with PLGA-NS

The upper Transwell^®^ inserts (polycarbonate membrane with a pore size of 8.0 µm, Corning Inc., Corning, NY, USA) were coated with BSA, collagen, and fibrin as described above. Then, the PH with CMR-PVA_200_-PLGA-NS were added onto the upper insert pre-coated and incubated for 30 min at 37 °C. After that, the inserts were washed with RPMI medium for three times and set on the bottom plate in which B16F10 cells (mouse melanoma cell line, RCB2630, provided by the RIKEN BRC through the National Bio-Resource Project of the MEXT/AMED, Japan) were cultured for 24 h. The B16F10 cells were cultured for further 3 days in RPMI medium supplemented with 10 vol% FCS and 1 vol% penicillin and streptomycin at 37 °C in a 5% CO_2_–95% air atmospheric condition. The medium was changed every day and the fluorescent images of B16F10 cells were taken by the fluorescent microscopy. Three images from each group were taken at random and analyzed using the computer program Image J. The Integrated Density of whole images was used as the fluorescent intensity for comparison.

To investigate the anti-tumor effect of PH on the B16F10 cells, the same procedures as described above were performed except for using PTX-PVA_200_-PLGA-NS for the preparation of PH. The number of B16F10 cells were counted every day by detaching with 0.25 wt% trypsin-containing 1 mM ethylenediaminetetra acetic acid (EDTA) solution (Nacalai Tesque Inc., Kyoto, Japan).

### 2.10. Statistical Analysis

The data were expressed as the mean ± standard deviation (SD). All the statistical analysis was performed using one-way analysis of variance (ANOVA) with a post-hoc Tukey–Kramer multiple comparison test; *p*-values less than 0.05 were considered to be statistically significant.

## 3. Results

### 3.1. Preparation of PLGA-NS

Various types of PLGA-NS were prepared by changing the preparation parameters, such as the types of surfactant polymers, PLGA concentration, stirring rate, and sodium chloride addition (Table 1). The zeta potential of PLGA-NS prepared was changed by using different types of surfactant polymers used. The size of PLGA-NS prepared became large as both the concentration of surfactant polymers or PLGA increased and the rate of stirring decreased. The addition of sodium chloride made the size of PLGA-NS large.

### 3.2. Platelet Activation by PLGA-NS

**Figure 1:**
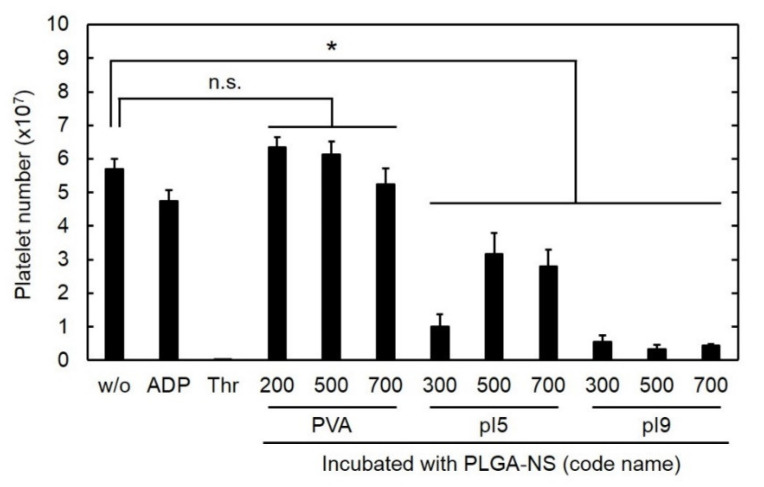
Number of platelets 30 h after incubation with PLGA-NS of different sizes prepared in the presence of various surfactants (PVA, pI5, and pI9). The ADP and thrombin (Thr) were used as agonists for platelet activation. n.s.: not significant. * *p* < 0.05: significant differences between two groups.

Figure 1 shows the number of platelets after incubation with PLGA-NS of different sizes prepared in the presence of various surfactants. The incubation with ADP or thrombin of agonists for platelet activation reduced the number of platelets although the extent of decrement for ADP was smaller than that for thrombin. In the same way, the number of platelets was significantly decreased by the addition of PLGA-NS prepared with pI5 and pI9. On the other hand, the incubation with PLGA-NS prepared with PVA did not affect the number of platelets. The PLGA-NS with a larger size tended to reduce the number of platelets to a great extent compared with smaller PLGA-NS. Based on the findings, the preparation condition of PVA_200_- (Table 1) was used in the following experiments.

### 3.3. Preparation of Drug-Loaded PLGA-NS

**Figure 2:**
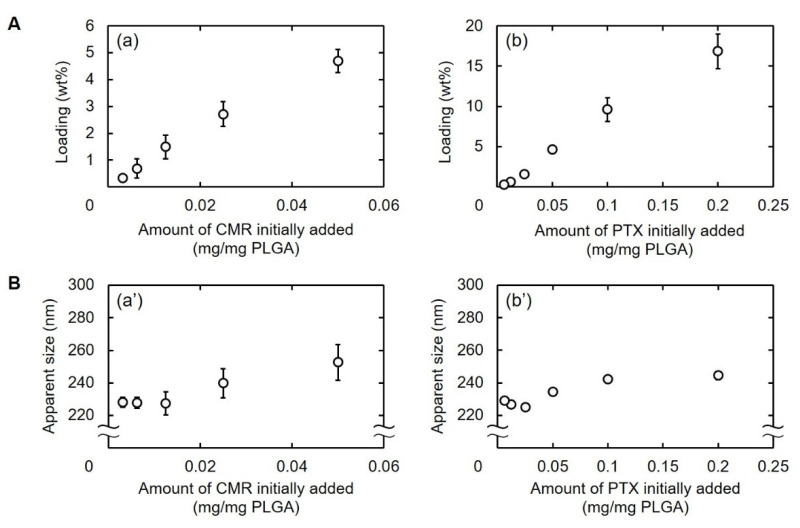
(**A**) Loading percentages of CMR (**a**) or PTX into PLGA-NS (**b**). (**B**) Apparent sizes of PVA_200_-PLGA-NS incorporating CMR (**a’**) or PTX (**b’**). Various amounts of CMR or PTX were initially added for nanospheres preparation.

Figure 2 shows the percentage of CMR or PTX loaded into PVA_200_-PLGA-NS and the size as a function of the amount of drugs added. The percentage of drug loaded increased with an increase in the amount of drugs initially added (Figure 2A). The size of PLGA-NS became large when prepared with large amounts of drug initially added (Figure 2B). The drug-loaded PVA_200_-PLGA-NS with an apparent size of around 240 nm were used for further experiments (Table 2).

### 3.4. Preparation of PH

**Figure 3:**
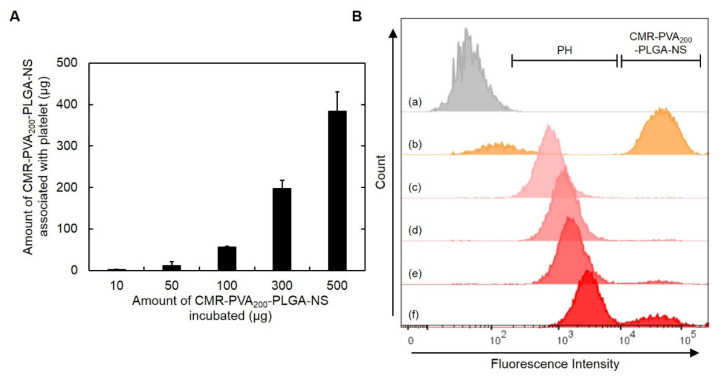
(**A**) The amount of CMR-PVA_200_-PLGA-NS associated with platelets 30 min after incubation with different amounts of CMR-PVA_200_-PLGA-NS. (**B**) Flow cytometric histograms of platelets (a), CMR-PVA_200_-PLGA-NS (b), or PH obtained by incubation with 10 (c), 50 (d), 100 (e), or 300 µg of CMR-PVA_200_-PLGA-NS (f).

Figure 3A shows the amount of CMR-PVA_200_-PLGA-NS associated with platelets. The amount increased with an increase in the amount of CMR-PVA_200_-PLGA-NS incubated. In the flow cytometric histograms, the peaks of PH shifted to a higher value of fluorescence intensity, while the peak attributed to CMR-PVA_200_-PLGA-NS was observed for the PH prepared by the association with 300 µg of CMR-PVA_200_-PLGA-NS (Figure 3B). Based on the results, the PH prepared with 100 µg of CMR-PVA_200_-PLGA-NS was used for the following experiments.

**Figure 4:**
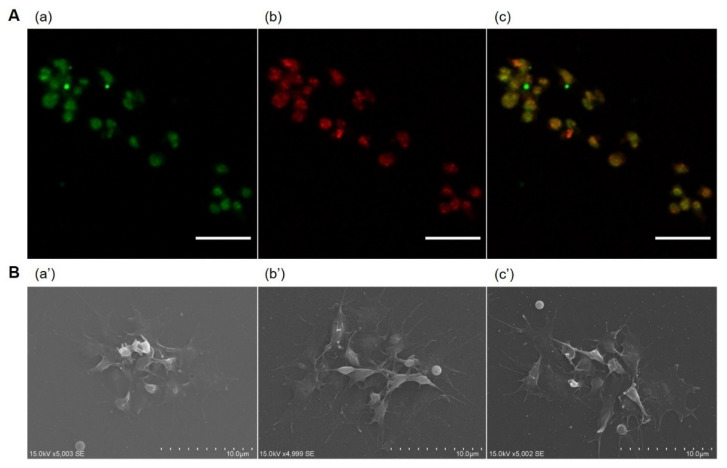
(**A**) Confocal microscopic images of PH incorporating CMR-PVA_200_-PLGA-NS. The PH were visualized by (**a**) CMR (green) incorporated into PVA_200_-PLGA-NS and (**b**) the staining with anti-CD41/61 antibody (red), then the merged image was obtained (**c**). Scale bar is 10 µm. (**B**) Scanning electron microscopic images of platelets (**a’**) or PH prepared by the incubation with PVA_200_-PLGA-NS (**b’**) or PTX-PVA_200_-PLGA-NS (**c’**) aiming at the observation of aggregating behavior on the glass bottom dish.

When observed by the confocal laser microscopy, the platelets were co-localized with CMR-PVA_200_-PLGA-NS (Figure 4A). From the SEM observation, no morphological change was observed for platelets or their hybrids prepared with PLGA-PVA_200_-NS or PTX-PVA_200_-PLGA-NS (Figure 4B).

### 3.5. Activation Behavior of PH

**Figure 5:**
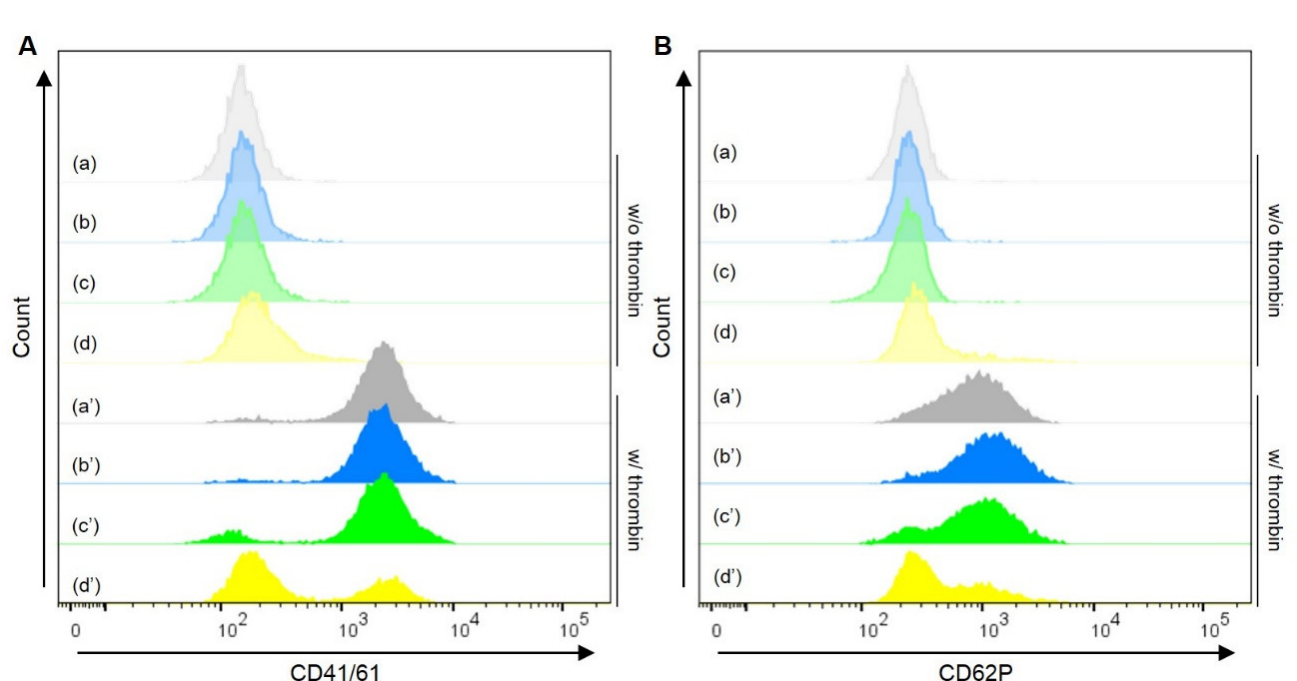
Flow cytometric histograms of platelets (a), PH with PVA_200_-PLGA-NS (b) or PTX-PVA_200_-PLGA-NS (c), or platelets incubated with PTX (d) under the treatment without (blurred colors, a–d) or with thrombin (plain colors, a’–d’). The platelets or PH were stained with CD41/61 (**A**) or CD62P (**B**). The amount of PTX in the PH (c, c’) was same as that incubated with platelets (d, d’).

Figure 5 shows the flow cytometric histograms of PH following the treatment with or without thrombin of an agonist for platelet activation. The PH prepared with PVA_200_-PLGA-NS and PTX-PVA_200_-PLGA-NS showed the similar histogram to the original platelets in terms of both CD41/61 and CD62P expressions. On the other hand, the platelet activation was inhibited by adding free PTX, followed by the stimulation with thrombin.

### 3.6. Affinity of PH for Collagen and Fibrin

**Figure 6:**
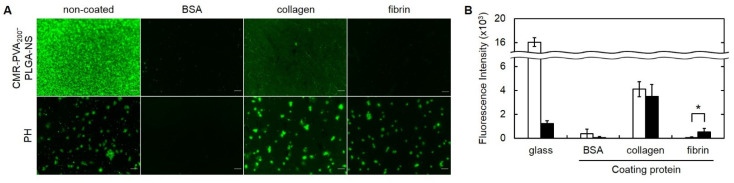
(**A**) Fluorescent microscopic images of CMR-PVA_200_-PLGA-NS or their PH adhered on the bare glass bottom dish or the dish pre-coated with BSA, collagen, and fibrin. (**B**) The fluorescence intensity 30 min after adding CMR-PVA_200_-PLGA-NS (□) or their PH adhered on the bare glass bottom dish or the dish pre-coated with proteins (BSA, collagen, or fibrin) (■). Six images were analyzed by using ImageJ 1.53. * *p* < 0.05: significant difference between two groups.

Figure 6 shows the adhesion behavior of CMR-PVA_200_-PLGA-NS with or without hybrid by platelets. The CMR-PVA_200_-PLGA-NS hardly adhered to the glass pre-coated with BSA, collagen, and fibrin. On the contrary, the PH with CMR-PVA_200_-PLGA-NS adhered to the glass or that pre-coated with collagen and fibrin. The adhesion of PH with CMR-PVA_200_-PLGA-NS was inhibited by the coating with BSA. The fluorescent intensity of PH with CMR-PVA_200_-PLGA-NS adhered on the fibrin-coated glass was significantly higher than that of CMR-PVA_200_-PLGA-NS alone, while no difference was observed for the collagen-coated glass.

### 3.7. CMR-NS Release from PH

**Figure 7:**
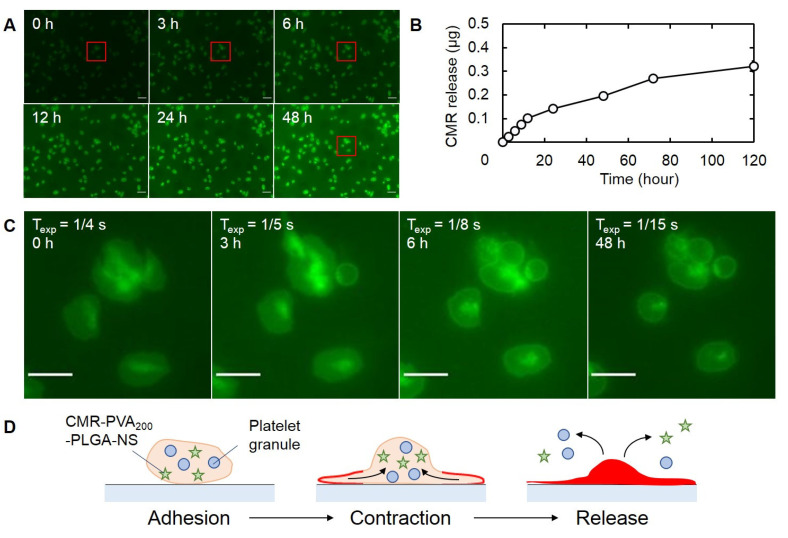
Time course of CMR-PVA_200_-PLGA-NS released from PH. (**A**) Time-lapse fluorescent microscopic images 0, 3, 6, 12, 24, and 48 h after incubation of PH. Scale bar is 10 µm. (**B**) Time profile of CMR release from PH. (**C**) Enlarged fluorescent microscopic images of red frame boxes indicated in (**A**). The values of T_exp_ indicates exposure time in the corresponding incubation periods. Scale bar is 5 µm. (**D**) The scheme of plausible mechanism on the release of CMR-PVA_200_-PLGA-NS from PH.

The release behavior of CMR-PVA_200_-PLGA-NS from PH was evaluated by time-lapse fluorescent microscopic images (Figure 7). When observed by fluorescent microscopy, CMR-PVA_200_-PLGA-NS were released from platelet into the culture medium with time (Figure 7A). This phenomenon was quantitatively confirmed by measuring the CMR released in the medium (Figure 7B). From the fluorescent observation at a high magnification, each platelet showed a rather brighter region inside initially and then the bright area decreased with time (Figure 7C).

### 3.8. Drug Delivery Profile of PH with PLGA-NS and In Vitro Anti-Tumor Effect

**Figure 8:**
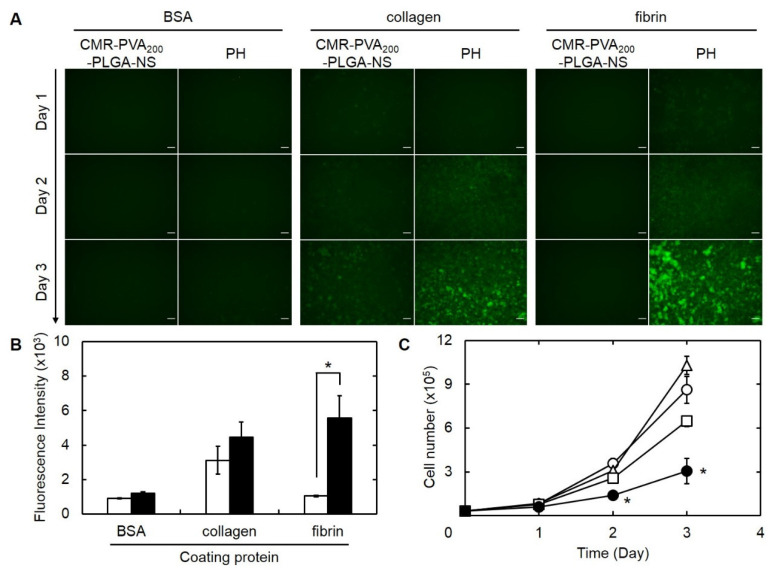
(**A**) Fluorescence microscopic images of B16F10 melanoma cells during 3 days of culture on the lower chamber inserted with the upper Transwell^®^ chamber pre-coated with BSA, collagen, or fibrin, followed by the culture with CMR-PVA_200_-PLGA-NS or their PH on day 0. Scale bar is 100 µm. (**B**) Fluorescence intensity of CMR in B16F10 cells 3 days after culture on the lower chamber inserted with the upper Transwell^®^ chamber pre-coated with BSA, collagen, or fibrin, followed by the culture with CMR-PVA_200_-PLGA-NS (□) or their PH on day 0 (■). * *p* < 0.05: significant difference between two groups. (**C**) Time profiles of B16F10 cells number cultured on the lower chamber inserted with the upper Transwell^®^ chamber pre-coated with fibrin, followed by the culture without (○) or with platelet (△), PTX-PVA_200_-PLGA-NS (□), and their PH on day 0 (●). The amount of PTX incorporated in each group (PTX-PVA_200_-PLGA-NS or PH) is 5.5 μg. * *p* < 0.05: significant difference against other groups at the corresponding time.

Figure 8A shows the fluorescence microscopic images of B16F10 melanoma cells cultured on the lower chamber after the addition of CMR-PVA_200_-PLGA-NS or their PH onto the upper Transwell^®^ chamber pre-coated with various proteins. Little fluorescence was observed for the addition of CMR-PVA_200_-PLGA-NS on the upper chamber pre-coated with BSA or fibrin. On the contrary, a bright fluorescence was observed for PH with CMR-PVA_200_-PLGA-NS in adding on the upper chamber pre-coated with collagen or fibrin. A significant difference of fluorescent intensity in the image analysis was observed between CMR-PVA_200_-PLGA-NS and their PH only when using the upper chamber pre-coated with fibrin (Figure 8B). Figure 8C shows the time profiles of B16F10 cells number cultured on the lower chamber after the addition of platelet, PTX-PVA_200_-PLGA-NS, or their PH onto the upper chamber pre-coated with fibrin. The increment of cell number was significantly inhibited by the addition of PH with PTX-PVA_200_-PLGA-NS.

## 4. Discussion

The present study demonstrates that a drug targeting system was achieved by the incorporation of drug into PLGA nanospheres and the subsequent association with platelets to form a PH. When applied for an in vitro tumor tissue model where tumor cells are separately combined with the insert pre-coated with the fibrin, the PH with PLGA nanospheres incorporating PTX enabled to suppress the growth of tumor cells through the specific adhesion to fibrin and the controlled release of the anti-tumor drug from the PLGA-NS.

For the efficient platelet-mediated drug targeting, it is highly required to associate drugs without any activation of platelets. It is well known that drugs sometimes affect the biological function of platelets through direct interaction. For example, the PTX itself suppresses the biological function of platelets [31]. Therefore, the incorporation of drugs into nanospheres without any induction of platelet activation is one of the practical ways to solve this issue. In the present study, first the effect of physicochemical properties of PLGA nanospheres (PLGA-NS) on the platelet activation was examined.

PLGA-NS with different sizes and surface properties were prepared by changing the type of polymer surfactant, the concentration of polymer surfactant and PLGA, and the stirring rate in preparation (Table 1). In the case of preparation in the PLGA-NS coated with PVA, the addition of sodium chloride was required to prepare the nanospheres with a large size. This can be explained by a high viscosity of the solution which affects the diffusion coefficient of organic solvents [32].

It was apparent from Figure 1 that the number of platelets did not change by adding PLGA-NS prepared with PVA, whereas it significantly decreased for PLGA-NS prepared with pI5 and pI9 gelatins. The platelet number decreased associated with the platelet aggregation induced by the activation as shown in the addition of ADP or thrombin. Therefore, this result clearly indicates that the surface property of nanospheres affects the platelet activation. It has been reported that nanospheres of metal, silica, lipid, and polymers activate the platelets in vitro or in vivo [33,34,35,36]. It is known that the glycoprotein VI of platelet membrane binds to the collagen’s repeated sequence of glycine-proline-hydroxyproline and subsequently forms a triple helix structure, resulting in the platelet activation [37,38]. Although both pI9 and pI5 gelatins are a denatured form of collagen, pI5 gelatin is processed with alkaline for a longer period of time compared with pI9 gelatin. It is likely that the structure of pI9 is similar to that of the original collagen compared with pI5 gelatin. Thus, we can say with certainty that the difference in the decreased extent of platelet number may be due to the secondary structure of gelatin used in the preparation of PLGA-NS. The decreased extent of platelet number also depended on the size of PLGA-NS prepared with PVA. This is because large PLGA-NS with more than 500 nm in diameter disrupt the plasma membrane of platelets, resulting in a decrease in the number after mixing [23].

The size of PVA_200_-PLGA-NS incorporating CMR or PTX tended to increase with an increase in the amount of CMR or PTX initially added (Figure 2). This is because a large amount of CMR or PTX with a water-insoluble nature located on the surface of PVA_200_-PLGA-NS, resulting in the aggregation of nanospheres to each other via the hydrophobic interaction [39].

The fluorescent spectroscopic analysis revealed that platelets could associate with CMR-PVA_200_-PLGA-NS while the extent depended on the initial addition of nanospheres (Figure 3A). In addition, the flow cytometric analysis revealed that a part of CMR-PVA_200_-PLGA-NS was not associated and present outside the platelet when the high amount of nanospheres was used for association with platelets (Figure 3B). This result clearly indicates that the maximum amount of nanospheres exists for the association with platelets. In addition, it was technically difficult to separate the excess nanospheres from PH only by centrifugation. Therefore, in the present study, 100 µg of PVA_200_-PLGA-NS was used to prepare the PH. Theoretically calculated, the number of CMR-PVA_200_-PLGA-NS associated with one platelet is estimated to be from 10 to 100. The association between platelet and CMR-PVA_200_-PLGA-NS was also confirmed by the confocal laser microscopy (Figure 4A). However, the detailed site of CMR-PVA_200_-PLGA-NS associated with platelets is unclear at present. The combination of a specific and stable labeling method of platelets and a microscopic system with a higher resolution may resolve this issue. It is apparent from Figure 4B that the presence of PVA_200_-PLGA-NS or PTX-PVA_200_-PLGA-NS did not affect the morphology and aggregation behavior of platelets. Taken together, it is clearly demonstrated that the association of platelets without activation was achieved by making use of the PLGA-NS prepared with PVA. The PLGA-NS prepared with PVA did not induce the platelet activation. One of plausible reasons to be considered is a weak interaction strength between the PVA on the PLGA-NS surface and the components of platelets surface. However, a certain degree of interaction should be required for the association of nanospheres with platelets. At present, there is no explanation to interpret this contradiction point.

It is reported that PTX inhibits the activation of platelets due to the stabilization of platelet cytoskeleton [26]. Similar behavior was observed in that the activation of platelets incubated with free PTX was inhibited even after adding thrombin of an activation agent (Figure 5A(d’),B(d’)). On the other hand, the activation after adding thrombin was observed for PH with PVA_200_-PLGA-NS incorporating PTX (Figure 5A(a’–c’),B(a’–c’)). This result clearly demonstrated that the incorporation into PVA_200_-PLGA-NS enables to maintain the biological function of platelets even after the association. The PH with PTX-PVA_200_-PLGA-NS after adding thrombin slightly inhibited the activation (Figure 5A(c’),B(c’)). It is possible that a small amount of PTX was released from PVA_200_-PLGA-NS inside the platelet, resulting in the inhibition of activation after even adding thrombin.

To demonstrate the feasibility of a targeting system based on PH for anti-tumor therapy, it is necessary to experimentally confirm the specific interaction of PH with target molecules and the consequent drug release. In this study, collagen and fibrin were selected as target molecules because the platelets have an inherent affinity for the molecules [40,41,42,43,44]. In tumor tissues, inflammation continuously takes place which often damages the blood vessel. With the vessel damage, collagen is exposed on the inner surface of blood vessels. Platelets have an inherent affinity for the collagen exposed [45]. In addition, the continuous bleeding in the tumor tissue often allows fibrin to deposit there. The platelets also adhere the fibrin specifically for blood clotting [46]. As expected, the PH specifically adhered onto the glass surface pre-coated with collagen and fibrin (Figure 6A). In addition, the quantitative analysis revealed that the PVA_200_-PLGA-NS showed a specific affinity for fibrin through the platelet association to a great extent compared with that for collagen (Figure 6B). Taken together, it is expected that the PH would be a feasible cargo for the tumor targeting of PVA_200_-PLGA-NS in vivo. Once the PH is localized in the tumor tissue, the drug should be released to act on tumor cells to exert the anti-tumor effects.

The time-lapse fluorescent imaging experiment (Figure 7A,B) revealed that the CMR as a model drug was gradually released from the platelets with time. In addition, in the fluorescent dynamics of CMR inside the platelet (Figure 7C), it was found that at first the CMR-PVA_200_-PLGA-NS was accumulated to one region to show a bright fluorescence, and then the shape of the region was changed, followed by a decreased fluorescent intensity. This observation suggests that the platelet actively released nanospheres out as illustrated in Figure 7D. It is well known that platelets release the platelet granules over activation [47]. Taken together, it is likely that the CMR-PVA_200_-PLGA-NS were released after the activation of platelets. However, it should be noted that all of the CMR-PVA_200_-PLGA-NS were not always released from the platelets during the experimental period. The illustration of Figure 7D shows one possibility and other behaviors will be considered.

There are various types of cells and extracellular matrix in the tumor tissue. In the present study, a Transwell^®^ system was used to mimic the in vivo environment of tumor tissue where the tumor cells are present separating from the extracellular matrix. The upper chamber pre-coated with fibrin or collagen and the lower chamber where the tumor cells are cultured were combined in the Transwell^®^ system, then the natural affinity of PH for collagen and fibrin and the anti-tumor effect of PH were investigated by applying the PH onto the upper chamber. The PH were specifically delivered to the tumor tissue separating from the upper chamber pre-coated with fibrin while the growth of tumor cells was significantly suppressed (Figure 8C). This result clearly indicates that the PH were specifically adhered onto the upper chamber through the platelet–molecule interaction. Then, the PTX incorporated into PVA_200_-PLGA-NS would be released from platelets, and the PTX released was delivered to tumor cells. In other words, the incorporation into PVA_200_-PLGA-NS allowed PTX to reduce the inherent toxicity for platelets and specifically deliver to tumor cells by the assistance of platelet inherent abilities for fibrin and release out of PTX from the PVA_200_-PLGA-NS uptaken. In the present study, where and how the PTX is released could not be clearly confirmed. In addition, the extent of PTX delivered to tumor cells was not quantitatively evaluated, either. These points should be investigated to make clear the detailed mechanism of the anti-tumor effect.

In future research, this platelet-mediated drug delivery system should be applied to in vivo experiments. It is well recognized that platelets have an affinity for the tumor stroma. Based on the research of “cancer stromal targeting (CAST)” therapy, an animal model of pancreatic cancers was considered to evaluate the anti-tumor effect [40]. In addition, it is known that platelets also have an ability to accumulate in inflamed vessels and tissues [48,49,50]. This platelet-mediated drug delivery system will be also applied to inflammatory diseases.

Authors should discuss the results and how they can be interpreted from the perspective of previous studies and of the working hypotheses. The findings and their implications should be discussed in the broadest context possible. Future research directions may also be highlighted.

## 5. Conclusions

By adjusting the parameters in preparation, PLGA-NS with different sizes and surface properties were prepared with surfactant polymers. The PLGA-NS prepared with PVA did not induce platelet activation which is a practical problem to be resolved in the construction of PH. The PVA_200_-PLGA-NS were expectedly associated with platelets by the simple incubation and separation. In addition, the presence of a PVA_200_-PLGA-NS incorporating drug did not affect any biological functions of platelets. As expected, the PH actively released PVA_200_-PLGA-NS incorporating drugs after they adhered to the fibrin-coated surface. The drugs released from platelets were specifically delivered to tumor cells and showed the in vitro suppression of tumor growth.

## Figures and Tables

**Table 1:** Preparation and physicochemical properties of PLGA nanospheres.

Code	Preparation Condition				Property						
	Surfactant (*w/v*%)	PLGA (*w/v*%)	Stirring Rate (rpm)	NaCl (mM)	Apparent Size (nm)			PDI ^a^	Zeta Potential (mV)		
PVA_200_	1	2	800	−	220	±	0.93 ^b^	0.047	−0.77	±	0.16
PVA_500_	1	2	400	500	470	±	11	0.17	−0.62	±	0.098
PVA_700_	4	4	400	1000	660	±	14	0.21	−0.56	±	0.11
pI5_300_	1	0.5	800	−	310	±	2.7	0.15	−10	±	0.87
pI5_500_	2	2	400	−	490	±	1.1	0.14	−7.3	±	0.41
pI5_700_	2	4	400	−	600	±	29	0.16	−7.8	±	1.1
pI9_300_	1	0.5	1200	−	290	±	1.8	0.1	−1.9	±	0.19
pI9_500_	1	2	800	−	460	±	11	0.15	−1.9	±	0.19
pI9_700_	1	4	400	−	670	±	21	0.16	−2.0	±	0.58

^a^ Polydispersity index, ^b^ Mean ± SD.

**Table 2:** Physicochemical properties of drug-loaded PLGA nanospheres.

Code	Apparent Size (nm)			Zeta Potential (mV)			Loading (wt%)
PVA_200_-PLGA-NS	220	±	0.93 ^a^	−0.77	±	0.16	−
CMR-PVA_200_-PLGA-NS	240	±	8.9	−0.51	±	0.18	2.7
PTX-PVA_200_-PLGA-NS	240	±	1.4	−0.59	±	0.097	9.6

^a^ Mean ± SD.
